# Trichobezoar effectively treated with direct endoscopic injection of Coca‐Cola: A case report

**DOI:** 10.1002/deo2.283

**Published:** 2023-09-25

**Authors:** Ryo Matsuoka, Saori Masuda, Satoshi Fujita, Naoe Akiyama

**Affiliations:** ^1^ Department of Pediatrics Fuji City General Hospital Shizuoka Japan; ^2^ Department of Pediatrics The Jikei Medical School of Medicine Tokyo Japan

**Keywords:** Coca‐Cola, endoscopy, hair bezoars, pediatric, trichobezoars

## Abstract

Trichobezoars (hair bezoars) are primarily observed in adolescent girls who pull their hair followed by its ingestion. Endoscopic removal of trichobezoars is challenging, and these masses often require surgical removal. Recently, although it has been reported that Coca‐Cola could effectively dissolve persimmon phytobezoars, it was ineffective in dissolving trichobezoars. We report a case in which Coca‐Cola was directly injected into a trichobezoar followed by successful endoscopic removal of the mass. A 9‐year‐old girl visited our hospital with abdominal pain and nausea, wherein abdominal radiography revealed a mass in the stomach. Her mother witnessed her pulling and ingesting her hair 6 months previously. An upper endoscopy was performed for diagnosis of the trichobezoar. Endoscopic removal of the mass was performed under general anesthesia following oral administration of Coca‐Cola at a dose of 100 mL thrice a day for 10 days. Initially, we attempted endoscopic extraction using grasping forceps and a radiofrequency snare. However, the bezoar could not be fragmented and did not pass through the cardia. Thus, Coca‐Cola was injected directly into the bezoar using a local injection needle, which facilitated the separation of the bezoar and allowed the grasping forceps to fragment it to a size that could pass through the cardia. Owing to the large size of the bezoar, we could remove 180 g of it without complications. The patient received psychological counseling after the procedure, to prevent recurrence. In conclusion, direct injection of Coca‐Cola was effective in the complete endoscopic removal of trichobezoars.

## INTRODUCTION

Trichobezoars (hair bezoars) are primarily observed in adolescent girls who pull and subsequently ingest their hair. Endoscopic removal of large trichobezoars is challenging, and these masses often require surgical removal. Recently, although it has been reported that Coca‐Cola could effectively dissolve persimmon phytobezoars, it was ineffective in dissolving trichobezoars.^1^ To date, no reports have specifically described the therapeutic effect of oral Coca‐Cola intake and/or endoscopic injection on trichobezoars. Herein, we report a case in which the endoscopic removal of a trichobezoar was successful following a direct endoscopic injection of Coca‐Cola.

## CASE REPORT

**FIGURE 1:**
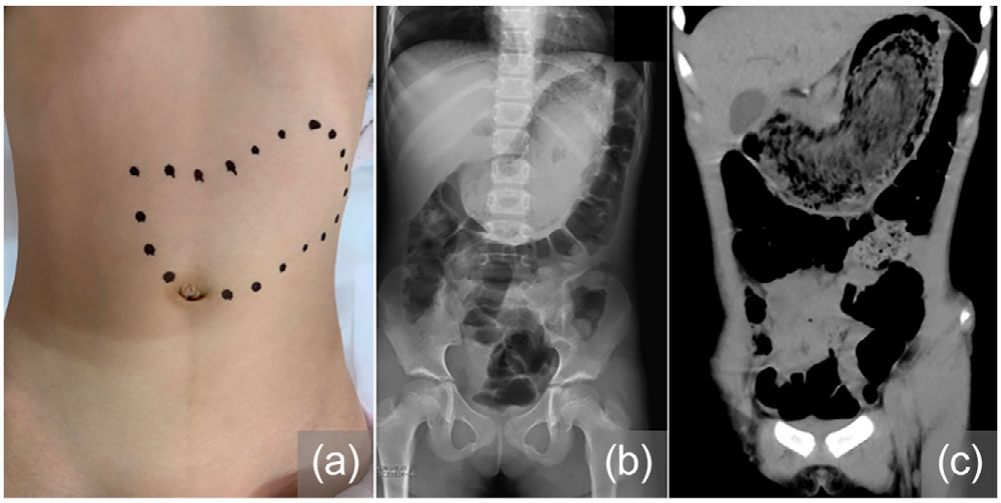
(a) A huge mass is palpated in the upper right abdomen. (b, c) X‐ray and abdominal computed tomography show a large gastric mass.

**FIGURE 2:**
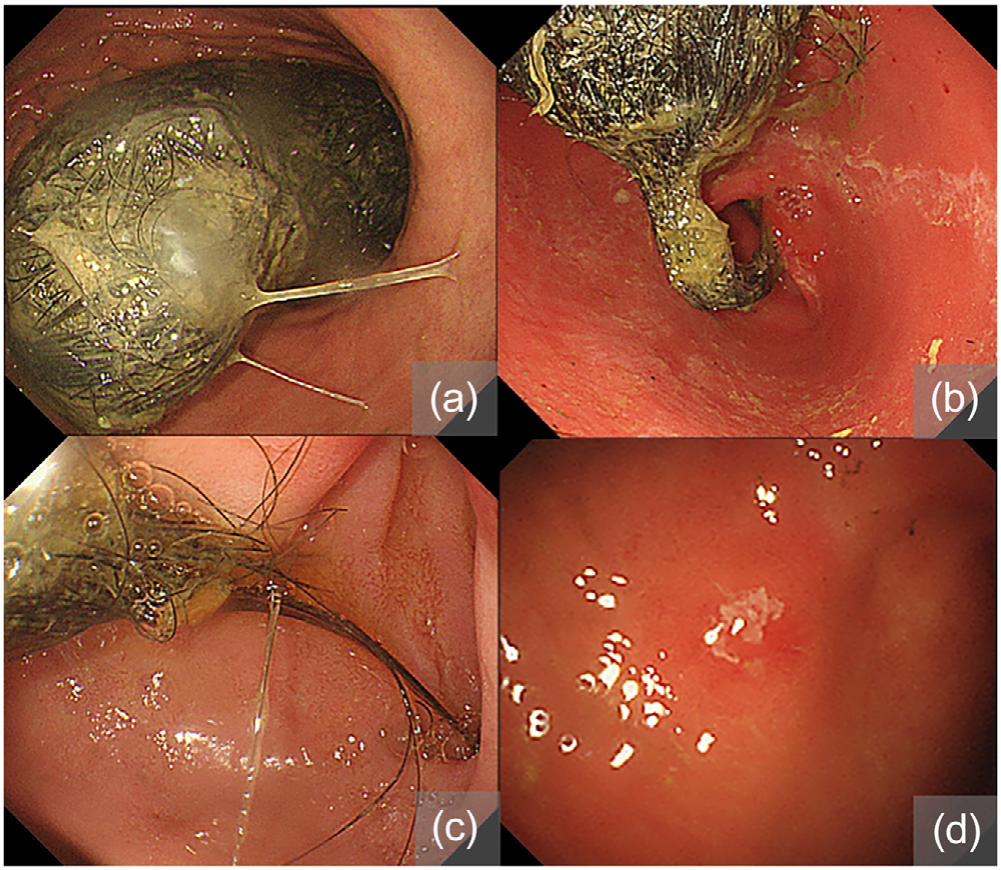
Endoscopic findings at first endoscopy. (a–c) A large trichobezoar is detected in the stomach extending beyond the duodenal bulb. (d) A small erosion is observed in the gastric angle.

A 9‐year‐old girl visited our hospital with abdominal pain and nausea. Physical examination revealed a fist‐sized mass in the abdomen (Figure 1a). Abdominal X‐ray and computed tomography confirmed a large mass (13 × 10 × 4 cm in size) in the stomach (Figure 1b,c). Her mother witnessed her pulling and ingesting her hair 6 months previously. There was no history of consuming tannin‐containing plants. Upper endoscopy was performed under intravenous anesthesia. A large quantity of hair‐entangled solid material was identified in the stomach (Figure 2a,b). Although the hair mass extended into the duodenal bulb, there was no indication of pyloric or intestinal tract obstruction (Figure 2c). A small erosion was observed at the gastric angle (Figure 2d). The patient was diagnosed with a trichobezoar and scheduled for endoscopic removal under general anesthesia at a later date. Following admission, the symptoms resolved, Moreover, due to the absence of endoscopic evidence of obstruction, oral administration of Coca‐Cola (Coca‐Cola Japan Company) was attempted until the scheduled procedure. Coca‐Cola was administered at a dose of 100 mL three times per day for 10 days prior to the procedure. Throughout the period of oral ingestion of Coca‐Cola, the patient received a nasogastric tube and intravenous omeprazole (proton pump inhibitor) and remained fasted, in which there were no signs of abdominal pain or other symptoms relapse.

**FIGURE 3:**
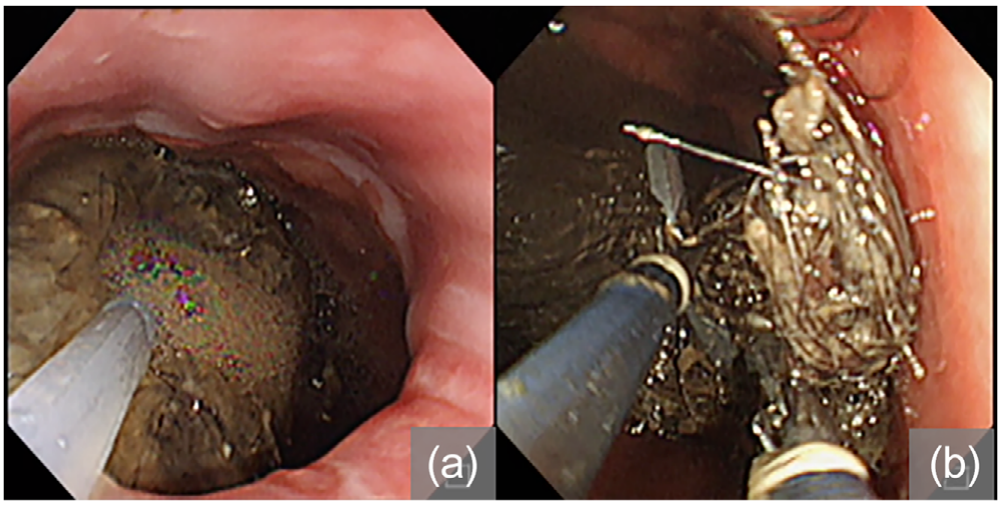
Endoscopic removal of the large trichobezoar. (a) Coca‐Cola is injected into the bezoar under endoscopy. (b) The bezoar is fragmented into small pieces using two grasping forceps.

**FIGURE 4:**
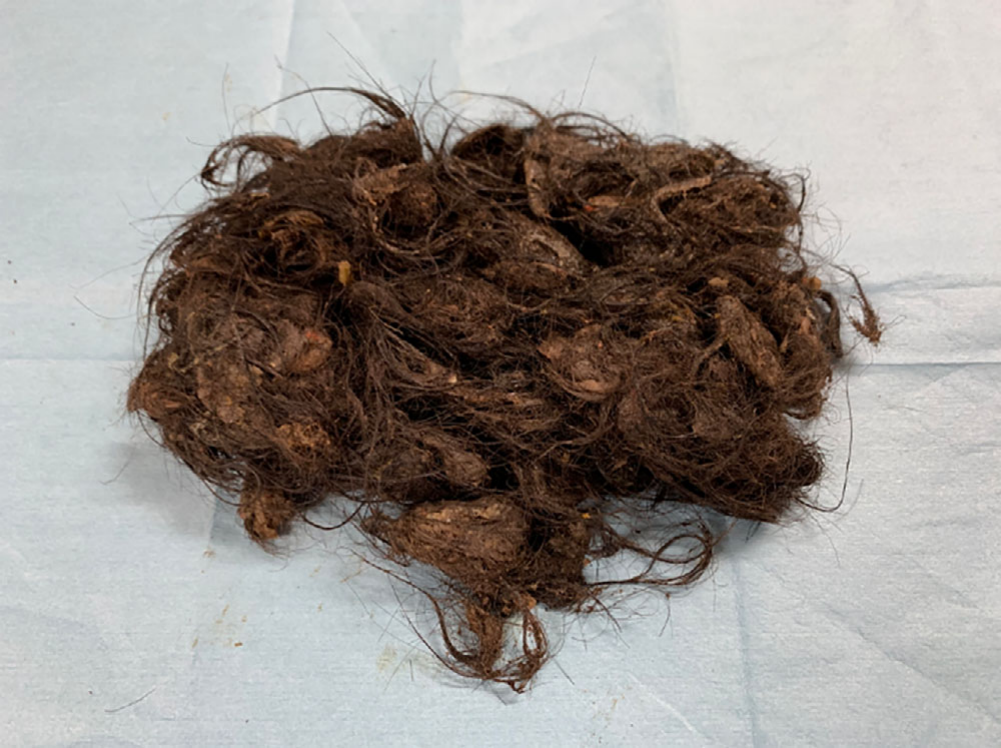
Trichobezoar specimen. The trichobezoar (total mass, 180 g) is completely removed during two endoscopic procedures.

A dual‐channel endoscope was used during the procedure (GIF‐2T240; Olympus). An over‐tube (MD‐48518; Sumitomo Bakelite Co., Ltd.) was inserted to prevent mucosal damage during endoscopy and trichobezoar removal. The condition of the trichobezoar was assessed after the oral administration of Coca‐Cola (100 mL three times per day for 10 days); apparently, no new X‐ray or endoscopic findings were noticed (Figure S1). Initially, extraction using grasping forceps (FG‐42L‐1; Olympus, Tokyo, Japan) and a radiofrequency snare (6183 Rotatable Snare Medium Oval 20mm; Boston Scientific Japan) was attempted. However, the bezoar could not be fragmented. Consequently, Coca‐Cola was injected directly into the bezoar using an injection needle (NM600/610; Olympus) and the bezoar gradually softened (Figure 3a). Coca‐Cola was carefully injected in small increments into the bezoar from multiple directions using a 25 mL syringe at approximately 20 mL per infusion to achieve thorough distribution from the surface to the interior of the bezoars. These bezoars were soft enough to be effortlessly punctured. Coca‐Cola was injected from the outer surface to the interior of the bezoar. Two grasping forceps were used to pull and divide the hair mass into pieces that could pass through the cardia (Figure 3b). Due to the large size of the bezoar, endoscopic removal was performed in two parts (Video S1). Finally, we removed 180 g of bezoar (Figure 4) without complications. Furthermore, the mucosal damage observed during the initial endoscopy showed improvement (Figure S2). In total, 500 mL of Coca‐Cola was used in a single procedure. The patient received psychological counseling after the procedure to prevent recurrence: no recurrence was observed 1 year later.

The patient's parents consented to the details of this case being published.

## DISCUSSION

Herein, we report the first case of complete endoscopic removal of a large trichobezoar using direct injection of Coca‐Cola. Oral Coca‐Cola treatment alone proved ineffective after comparing endoscopic changes before and after administration.

Although most bezoars are phytobezoars, trichobezoars are particularly common in adolescent girls and are characterized by a history of trichotillomania and trichophagia (hair loss and eating), which has been associated with emotional and affective disorders. Although phytobezoars are soft, relatively easy to crush, and soluble, trichobezoars are difficult to divide because long hair could be entangled, forming a large mass with food debris and fat.

In addition to lytic therapy, the treatment of bezoars is classified into endoscopic removal, laparoscopic resection, and surgical excision. Gorter et al. summarized the treatment of 108 trichobezoars in 2010 and reported a 5% success rate for endoscopic removal.^2^ According to recent reports, there has been an increasing prevalence of endoscopic removal of trichobezoars, with an estimated rate of approximately 17%.^3^ This can be attributed to advancements in endoscopic techniques and devices. Wang et al. reported that trichobezoars could be removed using a radiofrequency snare.^4^ However, in the present case, the division was not possible because the bezoar was too large and difficult to grasp with the snare. Hair and food residues were entangled with the hair, resulting in reduced electrical conductivity. Since the report by Gorter et al. and Lu et al., there have been reports of endoscopic removal of trichobezoars.^2^, ^3^ However, all of these were smaller in size than that in the present case,^2^, ^3^ suggesting that they were easier to grasp with snare and forceps.^5^, ^6^ Lu et al. indicated that successful endoscopic removal could depend on the size and morphology of bezoars.^3^ The removal of large trichobezoars appears to be difficult with an endoscopic procedure only.

Although typical dissolution treatment of bezoars uses digestive enzymes, in 2002, Ladas et al. reported that oral Coca‐Cola therapy was effective in the treatment of phytobezoars (plant bezoars).^7^ Subsequently, there have been reports indicating the effectiveness of oral Coca‐Cola therapy in bezoars, and in vitro studies have shown that Coca‐Cola tended to be more soluble than papain and cellulase in phytobezoars.^8^ In contrast, oral administration of digestive enzymes is ineffective in trichobezoars,^1^ and no cases have been reported in which trichobezoars were dissolved by oral intake of Coca‐Cola alone. In the present case, similar to previous cases, there was no significant X‐ray and endoscopic difference after administrating Coca‐Cola (3 L total) over 10 days orally. Additionally, there have been no reports confirming the efficacy of endoscopy evaluation of oral Coca‐Cola administration.

We encountered trichobezoars that were difficult to remove by endoscopy alone but could be removed endoscopically by injecting Coca‐Cola directly into the bezoar, resulting in softening of the mass. Ota et al. reported a case of tannin‐phytobezoars endoscopically removed with the administration and injection of Coca‐Cola.^9^ Gülerman et al. also used a combination of oral and direct endoscopic injection of Coca‐Cola to trichobezoars, the hair mass was difficult to divide and remove completely.^10^ The method of Coca‐Cola injection has not been described. However, in the present case, we injected Coca‐Cola in small increments onto the surface or inside the bezoar using an injection needle. This procedure was effective within a few minutes, resulting in easier removal of hair from the bezoar. The mechanism by which Coca‐Cola is effective in the removal of bezoars remains unknown. However, based on our experience, we suggest that the hair itself is not dissolved by Coca‐Cola; instead, food residues and fat components forming the mass are dissolved, facilitating endoscopic removal. Trichobezoars were not dissolved nor removed by oral Coca‐Cola administration or endoscopy alone, suggesting that direct injection of Coca‐Cola was effective.

In conclusion, direct injection of Coca‐Cola was effective in the complete endoscopic removal of trichobezoars.

## CONFLICT OF INTEREST STATEMENT

None.

## Supporting information

Figure S1 Initial and follow‐up abdominal radiograph findings before (a1,2) and after (b1, 2) administration of Coca‐Cola. White arrows indicate the bezoar. No obvious change is observed during both examinations.

Figure S2 Endoscopic findings after bezoar removal. (a, b) The bezoar is successfully removed without any complications. (c) The mucosal damage observed during the initial endoscopy demonstrated improvement.

Video S1 Endoscopic Coca‐Cola injection delivered directly into the bezoar. With two grasping forceps, before Coca‐Cola injection, the bezoar cannot be fragmented. After injection, the bezoar is divided into pieces that can pass through the cardia.
