# Supplementary figures and images for: Trichobezoar effectively treated with direct endoscopic injection of Coca‐Cola: A case report
Source: DEN Open. 2023 Sep 25;4(1):e283. doi: 10.1002/deo2.283 (PMC10518563; doi:10.1002/deo2.283)

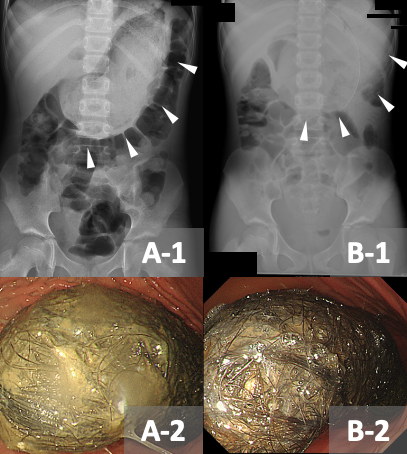

Supplement: Supplementary file 1 — Figure S1 Initial and follow‐up abdominal radiograph findings before (a1,2) and after (b1, 2) administration of Coca‐Cola. White arrows indicate the bezoar. No obvious change is observed during both examinations. [file DEO2-4-e283-s002.tiff]

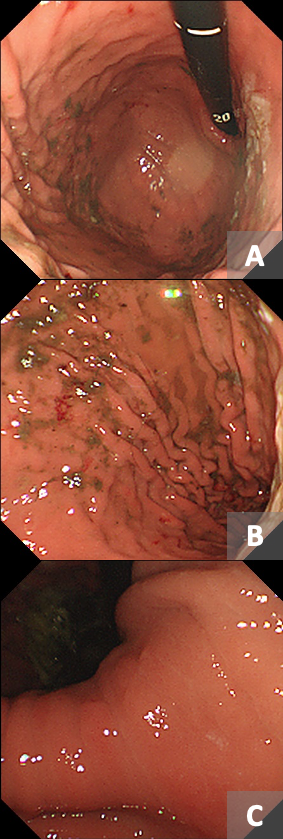

Supplement: Supplementary file 2 — Figure S2 Endoscopic findings after bezoar removal. (a, b) The bezoar is successfully removed without any complications. (c) The mucosal damage observed during the initial endoscopy demonstrated improvement. [file DEO2-4-e283-s003.tiff]
